# Socio-technical system analysis of responsible data sharing in water systems as critical infrastructure

**DOI:** 10.3389/fdata.2022.1057155

**Published:** 2023-01-04

**Authors:** Peter Hazell, Peter Novitzky, Steven van den Oord

**Affiliations:** ^1^Yorkshire Water UK, Cyber Physical Security Manager, Bradford, United Kingdom; ^2^Department of Computer Science, School of Computing and Engineering, University of Huddersfield, Huddersfield, United Kingdom; ^3^Department of Science, Technology, Engineering and Public Policy, University College London, London, United Kingdom; ^4^Avans University of Applied Sciences, Breda, Netherlands; ^5^University of Antwerp & Antwerp Management School, Antwerp, Belgium

**Keywords:** water systems, critical infrastructure, socio-technical system analysis, Capabilities Approach, datafication

## Abstract

Attention is increasingly focused on the protection of water systems as critical infrastructure, including subsystems of supply, sanitation, hygiene, and management. Similarly increasing consideration is paid to the growing role and impact of data on water systems and management. We explore key challenges associated with data-driven water systems as critical infrastructure. First, we describe the status of water infrastructure as a part of national critical infrastructure. Second, as this infrastructure increasingly relies on the constant flow of data from a huge variety, quality, and complexity of sensors, we provide a descriptive framework to map in detail the particular expertise needed across data-driven water management, applied to the UK water infrastructure as our use case. Third, through the framework of Capabilities Approach (CA) we analyze the specific challenges of data-driven water management, and argue that the current predominant narratives in the water infrastructure discourse have difficulties to effectively convey existing and emerging challenges. Fourth, we further demonstrate the widening gap between infrastructure services and consumer goods, arguing for increased convergence of the utilization of consumer data, and developing open data ecosystems.

## 1. Introduction

Leonardo da Vinci called water the driving force of nature (Kalen, 2019), and Earth is often called the blue planet because its hydrosphere occupies 71% of the planet's surface (Pidwirny, 2006). Since Earth formed, the amount of water on it has remained the same; comprised of 97% saltwater and 2% freshwater trapped in ice caps and glaciers, which leaves only 1% for humankind's needs (UNA-UK, 2021). Yet, over the past decades (clean) drinking water resources have dropped massively in terms of quality and quantity due to anthropogenic influences, such as that on climate change, making the availability of drinking water increasingly limited (Ahlström et al., 2021).

Since ancient Egypt, humanity has relied on data and monitoring of water levels to achieve large-scale water management (e.g., nilometer; Selin, 2016, p. 2359). The 3rd (digital) Industrial Revolution, which started in the 20th Century, witnessed large-scale data acquisition for control and automation in the industrial sphere (Rifkin, 2011). Despite this increase in the volume of data, analytics were restricted to toolsets such as Structured Query Language (SQL) (Vaidya, 2021) limited to processing structured data (Kulkarni and Harman, 2011a). This period largely predates the concept of open data (Chignard, 2013), so the ability to share any processed information that was available, even between systems of record within a single enterprise, was limited.

The digital transformation of our societies (the 4th Industrial Revolution, also called Industry 4.0, Floridi, 2014) offers opportunities to address water-related challenges. Water resources and systems are being digitalized, with digital infrastructures that include Internet of Things (IoT) devices such as sensors, smart appliances and homes, industrial sensors, inspection drones, and interconnected networks of embedded systems utilizing machine learning (ML) techniques. The collection and analysis of data on water resources and water systems may contribute in multiple ways toward a more sustainable and equitable use of water (UN, 2015b). Data-driven water systems promise to enable the (a) gathering of (better) insights on the supply and sanitation of water; (b) optimization of water resources; (c) improvement of safeguards for water protection; and (d) creation of economic value as data is an intangible economic asset. For instance, the concept of a virtual geographic water footprint makes it possible to assess how some countries significantly influence water consumption and pollution across the globe through the import and production of goods and services that require water (Mekonnen and Hoekstra, 2011; Mekonnen et al., 2015).

Legally, the United Nations (UN) Resolution A/RES/64/292 acknowledges clean drinking water and sanitation as essential to realizing all human rights (UN, 2015a; Kalen, 2019). In Europe, EU (2012), Art 37 refers to the principle of sustainable development, and the new Drinking Water Directive (2020/2184) obliges EU member states to protect human health from the adverse effects of any potable water contamination by ensuring that the water is wholesome and clean, as part of their respective national legislations until 2023 (EC, 2021). During the consultation period, the European Economic and Social Committee's opinion expressed opposition to water privatization (EESC, 2018), while some EU countries introduced additional water-protecting legislation [e.g., Austria (Österreich, 2019); Slovakia (Balogová, 2014)].

Kalen (2019) concludes that while water rights enjoy wide generic consensus, the enforceability of such rights does not go beyond aspirational pronouncements and is therefore highly dependent on the legal systems of the individual countries. The prominent position of water in (inter)national public policy has recently been confirmed in the UN Sustainable Development Goal (SDG) n.6, which recognizes the need for nations to ensure access to water and sanitation for their citizens as a global societal challenge (UN, 2021). SDG6 aims to provide universal access to and sustainable management of clean and affordable water by 2030 globally, as fresh water in sufficient quantity and quality is essential for all aspects of life, including sustainable development, food security, health, and the reduction of poverty (UN, 2018).

Yet, over 800 million people continue to lack basic water services, and 2.1 billion experience difficult access to drinking water services in their living premises (UN, 2018). Increasingly scarce water ecosystems are under continuous pressure from industrial activities (critical) infrastructure development, pollution, and resource extraction. Such deteriorating ecosystems can cause (armed) conflicts and consequently the displacement of large populations (UN, 2018), while rising average temperatures may further aggravate the intensity of droughts and water scarcity—not limited only to the most deprived regions of the world (IPCC, 2018, 2019; Environment Agency, 2022). Environmental concerns and impacts of climate change due to global warming, together with increasing global populations, drive further debate about the need for greater protection of water systems and water as a basic need. In tackling all these challenges, the digitalization of water management will play a crucial role.

Against this backdrop, our objectives with this contribution are: first, to describe the status of water infrastructure as a part of national critical infrastructure. Second, this infrastructure increasingly relies on the constant flow of digital data from a huge variety, quality, and thus complexity of sensors and actuators. This creates challenges from the viewpoint of required expertise, risks, and benefits, as well as unforeseen consequences. Third, we analyze these through the framework of the Capabilities Approach (CA) and argue that the current predominant narratives in the water infrastructure have difficulties in conveying the existing and emerging challenges (e.g., securing the human right to drinking water; climate emergency). Following detailed analysis, we further demonstrate the widening gap between infrastructure services and consumer goods, arguing for increased convergence of the utilization of—currently wasted—consumer data and the development of open data ecosystems.

## 2. Water systems as data-driven critical infrastructure

### 2.1. Data and modern infrastructure

Information used in connection with water manifests itself in many forms ranging from the basic (animals' knowledge of rivers, water holes, etc.) to the complex datasets used to verify hydraulic models. Given this variability in use and the complexity of datasets in this domain, for any analysis to be meaningful, consideration must be given to an analytical framework against which conclusions can be formed.

Before these considerations are addressed, it is first necessary to understand the difference between data, information, structured and unstructured data, and knowledge; and the relationship between machine learning (ML) and the cyber-physical environment.

It has been estimated that around 80% of all current datasets are unstructured (Shilakes and Tylman, 1998; Taleb et al., 2018). Kitchin (2014) defines data as “raw material produced by abstracting the world into categories, measures, and other representational forms—numbers, characters, symbols, images, sounds, electromagnetic waves, bits—that constitute the building blocks from which information and knowledge are created” (Kitchin, 2014, p. 1). Consequently, unprocessed data is of little value in any decision-making process without being converted into information first—the corollary to this being that there is a distinction between sharing data and sharing information.

This reliance on information and knowledge (i.e., awareness or familiarity gained by experience; Fowler, 1995) in the decision-making process raises four further questions: (1) where does the underlying data originate from; (2) how is it processed into information; (3) how is the knowledge acquired applied; and (4) what is the impact of these transformation processes on water management?

The first step in addressing these questions is to define what is meant by “water management” in terms of scale and domain. In the context of this paper, water management encompasses anything that impacts water availability and quality, comprising domains such as catchments (including rivers, groundwater, and impounding reservoirs); clean treatment; clean distribution; waste collection; waste treatment; flood management; pollution control; weather forecasting, etc. Similarly, this paper is not confined to the type of data or information exchanged, be that: conferences and industry bodies exchanging best practices; data and information gathered and used by water companies to control and operate their assets; information exchanged between commercial and governmental organizations (e.g., flood management); water resource management (regional, national, and international); or any other form.

Likewise, the scope of this paper has no restriction on the scale of information shared, including everything from the civil public verbally communicating the location of the nearest water hole to the large, sophisticated datasets described as “big-data” (Raheem, 2020). Data can be represented in nature, implied, derived, stored, and recorded in analog or encoded in digital form (Kitchin, 2014). This distinction serves as an important reminder that data exists outside of the digital domain and may be collected manually even if it is later inputted by hand into a computer system. In addition to manual collection, data representing the characteristics of the real world may also be collected automatically by a process known as sampling (Kutsanedzie et al., 2016), which converts physical phenomena (levels, pressures, temperatures, images, sounds, etc.) into a digital representation of those phenomena (either as a binary number or true/false logic state) (Manganaro, 2014). In data logging applications, it is common practice to attach a time stamp to each sample, creating what is termed “time-series” data that can be stored and replayed to reproduce the system's behavior over time (e.g., a historical trend of the discharge flow, hence consumption, from a service reservoir). Irrespective of how data is collected (manually, audio recordings, samples, or time-series) for processing purposes, it is divided into one of three categories (Raheem, 2020): (a) structured (linked by regular fields—e.g., relational databases); (b) semi-structured (linked by metadata—e.g., OPC-UA's Address Space; OPC, 2017); (c) and unstructured (no obvious pattern—e.g., images and audio recordings).

Historically, it has always been assumed that humans are better than machines at recognizing patterns in unstructured data such as images and sound (e.g., the ability to recognize water by sight and ear) but less so for the repetitive processing of structured data (Özkiziltan and Hassel, 2020). However, with advances in ML, computers are increasingly used to identify patterns in unstructured data (Kulkarni and Harman, 2011b) (e.g., supplementing teams of humans with listening sticks by identifying leaks from audio files collected by acoustic loggers in drinking water networks; “Acoustic Ears' to Listen for Leaks, 2019”). This field of ML directed at large unstructured datasets is termed deep learning. It is a field in its own right, based on techniques such as neural networks, as opposed to those statistical tools such as linear regression used in the more established processing of structured data (Kulkarni and Harman, 2011a).

The potential benefits of using increasingly large datasets collected through IoT and Industrial Internet of Things (IIoT) devices (Lesniewska and McCann, 2019; Lindley et al., 2019) together with the increased use of deep learning, does raise questions of accessibility for regions lacking the infrastructure or technical expertise to support the technologies.

The example of the civil public above is informative as it sets a lower bound of system complexity that is both intuitive and understandable. The task of defining an upper bound is more challenging as it is dependent on several factors (e.g., sector, technology, uptake of big-data, data type, and application of ML), which together create many combinations. Kitchin and McArdle (2016) defined seven traits that distinguish big data from other datasets. Of these traits, velocity and exhaustivity are the most important (Kitchin and McArdle, 2016).

For our analysis, to circumvent this complexity, the upper bound is set in the following section through a case study based on the United Kingdom water industry, which also gives tangible examples of the origins of data, how it is processed into information and how it is applied to water management. Although limited to a single, regionally constrained sector, this description provides a foundation upon which other use cases can be compared. However, before embarking on this avenue, it is necessary first to define the different types of data and the methods to convert them to information.

### 2.2. Setting the upper bound of system complexity—The United Kingdom water industry

**Figure 1:**
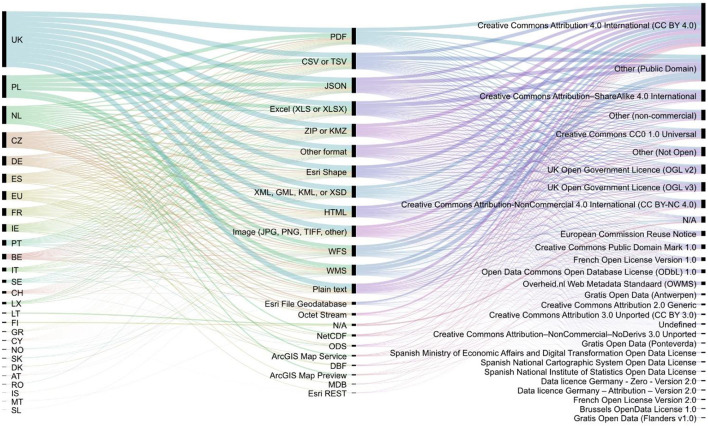
Data Europa.eu overview of water open datasets in 2021.

To concretize the overlaps between data-driven infrastructure and water management, we focus particularly on the UK Water Industry as a case illustration to set the upper bound of data complexity as follows:

- In contrast to other process industries (e.g., Power, Oil, Gas, etc.), which process their main asset independently, the large UK water companies are highly reliant on data to centrally manage both production and distribution asset types as an integrated system.
- The UK Water Industry relies on data to manage their Clean and Waste businesses as a unified enterprise, along with non-regulated businesses such as energy from waste.
- The recent focus by UK water companies on innovative technologies across Clean and Waste (evidenced by numerous references to data and technology in their literature such as Thames Water, 2020; Yorkshire Water, 2020a; “Anglian Water First Water Company in UK to Trial New Leakage Tech in Live Water Mains”, 2021) demonstrates the scale and investment in this area.
- The process of converting data into information requires investment in IT systems of record (Gorelik, 2019), ML (e.g., Water Breakthrough Challenge 1; OfWat, 2021a), and agile delivery techniques (APM, 2022).
- The UK Water companies are regional monopolies and, therefore, more able to share good practices than other more commercially competitive industries—evidenced by the creation of formal links such as Telecommunications Association of the UK Water Industry (2017); Water UK (2018), etc.; and less formal working groups such as the I4.0 initiative that meets quarterly to discuss issues of common interest in the Operational Technology (OT) sphere (including Digital Convergence, IIoT, advanced analytics and ML).
- Access to these bodies and groups gives the authors a reliable insight into how data is collected, processed, and used by the large UK water companies, from which the following generic model can be established with confidence.
- Our analysis of the available open datasets on the official portal for European data^1^ highlights that at the end of 2021 more than 17,000 open datasets exist on water, provided in more than 20 various formats, under more than 30 various types of licenses (Figure 1). The UK has contributed the largest amount, with more than 5,000 open datasets on water.
- The main motives to publish open datasets in the UK's water management services are (NWG, 2021; OfWat, 2021a; Yorkshire Water, 2021):

- Cross-industry dataset provision for independent data scientists;
- Improved standardized data quality, collection, and maintenance of datasets (e.g., pollution, consumption, resource, leakage, bio-resources);
- Increased ability to collaborate with local communities and digital developers on innovative solutions for water-related problems (e.g., through merging with other relevant datasets or gaining deeper insight *via* research);
- Collaborative validation of proof-of-concept solutions;
- Development of new business models and services;
- Enhanced customer experience and improved transparency toward the customers.

**Figure 2:**
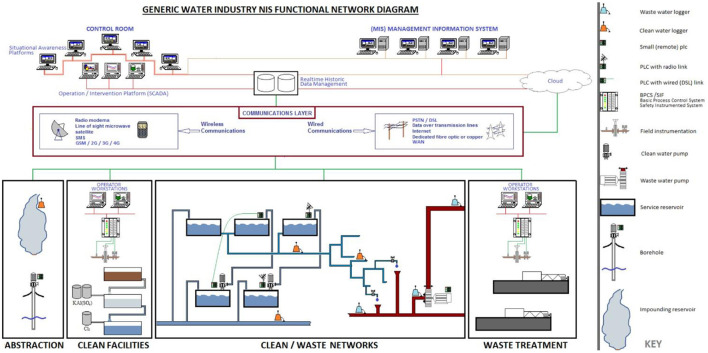
The collection and use of data by a generic large UK water company—based on inside industry knowledge and information acquired from industry groups. The bottom of the diagram shows the field layer connected to the IT layer (at the top) *via* a telemetry layer.

Figure 2 demonstrates the operational structure of a typical large UK water company running an integrated clean and wastewater operation, including both production and network assets. The bottom of the diagram shows (from left to right) how water is abstracted, treated, and the waste collected and treated. Although this diagram shows a closed system, it must be noted that this is only one part in a much larger system of water management encompassing the control of impounding reservoir levels used to reduce flooding (Yorkshire Water, 2020c), data being shared with the Environment Agency for regulatory purposes (Environment Agency, 2014), the dependence of the operation of the system as a whole on weather data, etc.

Traditionally, data at this bottom level of the diagram has been processed by three basic equipment classes. The first (data loggers) take real-world measurements by sampling sensor data, time stamping it, converting it to time series data, and transmitting the results to a central system of record for processing “(Data Loggers Data Acquisition, 2022)”. In Figure 2, data loggers are used throughout the network to measure physical parameters such as pressure, flow, and level data for various purposes, including resource management, catchment management, leakage detection, water quality, and pollution control.

The second class comprises automation equipment [typified by, but not limited to, Programmable Logic Controllers (PLCs)] which sample real-world parameters but primarily for control and automation of the process (Love, 2007). This process control class has two functions: (a) operating pumps and valves in response to changes in process conditions; and (b) providing data and information to operators [so they can intervene directly in the running of the process, operational support (materials ordering, maintenance, etc.)], and for managers to aid the running of the enterprise as a whole.

There is no single underlying technology associated with the third class yet, defined by the ability to take consecutive samples at a fixed frequency to record and process unstructured data such as audio, moving images, vibration data, etc.

The UK water industry relies on the coverage of sophisticated technologies mostly available in developed countries to harvest this data, ranging from simple Public Switched Telephone Network (PSTN)^2^ through to 5G and satellite. The dated PSTN is gradually replaced both in the information technology (IT) and OT domains with advances in (a) data acquisition using cheap IoT and IIoT sensors and improved communications coverage (e.g., 5G, Sigfox, NB-IoT; Sigfox, 2019; Thales, 2020; UK Government, 2020); (b) cloud service and Fog devices (Lesniewska and McCann, 2019) offering advanced analytics based on machine learning and unstructured data both centrally and at the edge (Lindley et al., 2019); (c) open systems using Application Programming Interfaces (APIs) (IBM Cloud Education, 2020); and (d) open protocols such as OPC-UA (OPC, 2017). The trend of using big data as a resource to improve efficiency in an industrial context represents the shift to Industry 4.0 (Schwab, 2017; Suri, 2018).

Similarly, the capacity to process and utilize this data depends on resources (both technological and workforce skills) not available in many countries. For instance, traditionally leakage detection relied on reports by members of the public and teams of employees looking and listening for evidence of leaks. Following the drought in 1995 the UK regulator tightened restrictions on permitted leakage (OfWat, 2015), which saw an immediate growth in the pressure and flow loggers used for this purpose. Initially, this information was processed by skilled teams of employees supported by the traditional database and geolocation tools. However, over time the range and sophistication of techniques have improved, and water companies are using a range of new tools, including acoustic loggers (Shrestha, 2019), drones (WADI Horizon, 2020), and even sniffer dogs (Mcallister, 2020).

This, in turn, has created a growth in the three data categories defined earlier, which has been matched by an increased reliance on traditional ML (to analyze existing structured and semi-structured data—e.g., time-series and geo-locational data). Furthermore, the introduction of deep learning allows one to gain further insights into these existing datasets while also analyzing the new unstructured data, such as audio files acquired from acoustic loggers.

This level of analytics requires a modern infrastructure, such the hybrid model illustrated in Figure 2, comprising a mix of company-owned data-centers (“on-premises”) and Cloud services. The UK water industry is thereof increasingly adopting those services normally associated with the Cloud (Software-as-a-Service [SaaS], Infrastructure-as-a-Service [IaaS], and Platforms-as-a-Service [Pass]). In addition, the UK water industry venturing into: Data-as-a-Service [DaaS^3^]; and Cloud tenancies such as Siemens' MindSphere (Siemens, 2021) and GE's Predix (Digital, 2022) platforms.

Figure 2 also shows how UK water companies use enterprise-level Supervisory Control and Data Acquisition (SCADA) systems to monitor and control assets remotely, in real-time, based on sample data collated in real-time databases, while storing time series data in historical databases for further analysis. The OT data collected in this way is blended with other data and information (e.g., geo-locational, customer data, weather data, etc.) stored either on-premises or in the Cloud to assist decision-making when operating assets in real-time (situational-awareness), or to support back-office functions such as scheduling teams of workers to repair leaks, clear sewer blockages, etc.

Returning back down the technology stack, this same model also applies to the Clean production, and Waste treatment facilities, the difference being that these assets are more complex, collect larger volumes of data, require a higher level of automation, and are controlled locally by operators using site SCADA systems.

Despite the restrictions placed on this case illustration (limited to a single, regionally constrained sector in a developed, island nation requiring little international cooperation in the management of resources and subject to a regulatory framework unique to that environment) it does describe the variety of data and information available, across all water management sectors and nations assuming the existence of the necessary, infrastructure and skills base required to support them.

The presented case illustration of the UK water infrastructure highlights the absence of the associated IT and OT engineering/technical expertise with the pressing societal challenges related to the human right to water, and the present climate emergency. In the next section, we approach the convergence of data and water infrastructures from a higher-order descriptive framework, which is based on innovation stages. We aim to investigate the reflection of societal and normative challenges in the current conceptualizations of data-driven water infrastructure systems.

### 2.3. Mapping the complementariness of data and water infrastructures—An analytical framework

Data-driven water management usually combines big data, Digital Twins (Verma, 2020), and Strategic Data Platform (i.e., the ability to create agile solutions enabling the blending and sharing of information derived from core systems of record; Gangopadhyay, 2021). Together they improve the management of clean and wastewater operations. These same systems generate information that partners can share to help manage regional issues such as flooding and other civil defense incidents.

**Figure 3:**
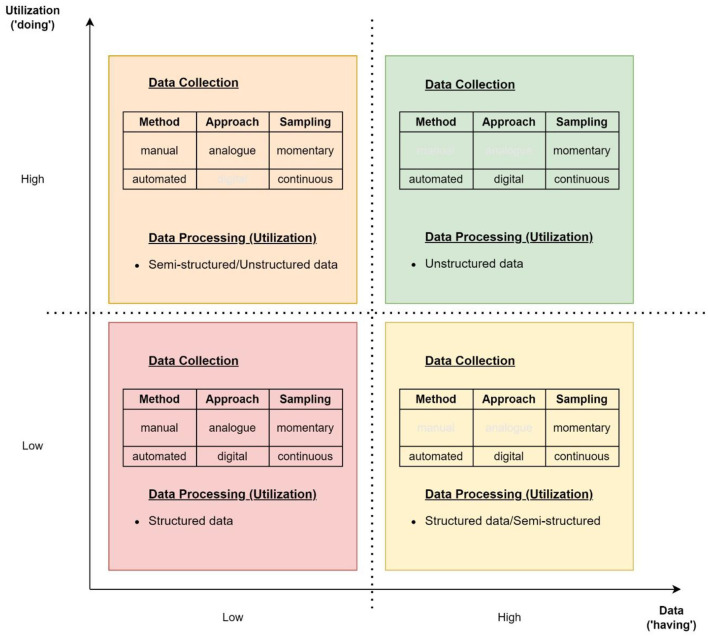
Mapping data onto the 2 × 2 matrix used later to analyze outcomes in water management, based on the four industrial revolutions—showing how the transition between the digital collection of sample and continuous data and the ability to process unstructured data may be used to define convenient metrics. Grayed out categories are non-applicable fields in the given section of the quadrant descriptive model.

The four stages of industrial revolutions provide a convenient classification system to align the continuum of data collection and usage defined above (Section 2) for further analysis. This analytical framework assigns abstracted facets of data usage to four quadrants of a matrix according to data collection and utilization (Figure 3).

The transition from low to high for the *x*-axis (data collection) aligns with that for the 2nd and 3rd Industrial Revolutions—with low collection typified by manual and analog techniques and high by the move to automated digital sampling of physical (real-world) parameters. The term “manual” in this context includes data collection in a digital form requiring a high degree of human input (e.g., writing, handheld measuring). Therefore, leakage detection derived from piloted drones (WaterJournalUK, n.d.) falls into the low category, whereas similar data collected by automated satellites are classified as high.

The *y*-axis (data utilization) is best divided based on the ability to analyze structured and unstructured data sets—i.e., broadly divided between the 3rd and 4th Industrial Revolutions—manual and semi-automatic data processing of the 1st and 2nd Revolutions was best suited to structured data processing along with the highly structured sample and time series data associated with the 3rd Industrial Revolution.

Our approach is predicated on the understanding that no single use case or domain inhabits just one of the four stages of industrialization. Still, the relevant quadrant can be identified based on the degree of adoption of technology, as it relates to a specific argument. For instance, nowadays most countries have access to satellite information enhanced by ML (despite not having the technology to derive it themselves). In contrast, countries with access to the full gamut of data gathering resources still resort to some manual recording of data (even if it is later inputted into a computer system). Despite both using the full range of available data collection and analytical methodologies, it is the highly industrialized countries who adopt the most up-to-date techniques at scale, and are therefore better able to benefit from the ability to share data.

Mapping the innovation stages to this descriptive framework, it is apparent there is a tendency to increase both the quantity and the quality of data collected to facilitate more efficient utilization of these for service maintenance. However, this shift is not even across the operational and information systems, while they reflect normative and societal challenges only indirectly. In the following analysis, we, therefore, highlight the need for and a proposal of a normative framework that can better facilitate the explicit reflection on societal challenges facing present and future water infrastructures.

### 2.4. Real-world application: The UK water management ecosystem

**Figure 4:**
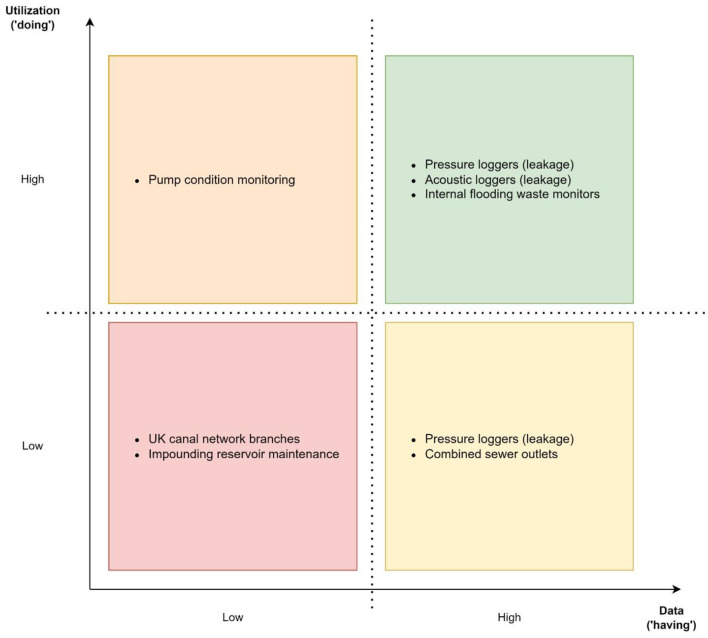
Real-world applications of data collection and analysis applied to water management.

To apply this descriptive-analytical framework, we explore a real-world application mapped onto the 2 × 2 matrix in Figure 4, explicating it by the UK water management ecosystem (e.g., UK's data-driven water management is exemplified by Yorkshire Water's Smart Networks Pilot; Yorkshire Water, 2020b).

The matrix delineates water management through data ownership and utilization, following the paradigms present at the stages of industrial revolutions. As the stage of industrial revolutions continue, both the amount and processing of data increase. Moreover, the shift from structured/semi-structured toward unstructured data occurs during this development.

#### 2.4.1. UK canal network

##### Classification: Low data collection/low data utilization

The UK Canal and River Trust's central control room is connected to a network of 600+ strategically deployed canal monitoring stations (CanalRiver Trust, 2019)—for comparison telemetry endpoints for a typical large regional UK water company are numbered in the low tens of thousands. It is, therefore, reasonable to conclude from this and other evidence (e.g., it is unlikely that the cost of automated data collection could be justified for the weekly data collection of reservoir levels undertaken by the trust; CanalRiver Trust, n.d.) that water management of the UK canal network is primarily based on manual data collection and simple analytics.

#### 2.4.2. Impounding reservoirs maintenance

##### Classification: Low data collection/low data utilization

The failure of impounding reservoirs poses a considerable risk to the public (Mauney, 2020). As such, they are regulated by the UK government who mandate a raft of inspections—although wide-ranging from a data perspective, they largely involve manual measurements [e.g., using and surveying methodologies to detect movement in dam walls and inspections of the physical asset (Environment Agency, 2021)]. The data collected is then subjected to traditional modeling methodologies.

#### 2.4.3. Leakage detection

##### Classification: High data collection/low data utilization

UK water companies use pressure and flow data loggers (numbered in the thousands) to detect leaks across their drinking water networks. The theory is simple, given that pressure in a pipe is a function of height and that any deviation from that profile must equate to a leak. However, similar variations are more likely to result from legitimate use.

Historically this structured (time series data) harvested from the loggers has been, and continues to be, processed using traditional database tools and teams of experts analyzing the data using human experience to identify leaks.

##### Classification: High data collection/high data utilization

Although the use of human experience to analyze pressure and flow data is still the predominant model, it is being increasingly augmented by machine learning looking for similar patterns as the human element, and in some cases looking for the patterns of activity that might result in a future leak.

In addition to these back office “leakage teams” (whose job it is to identify leaks and schedule their repair) the central control room also makes use of this data to identify major leaks in real time (e.g., bust mains) that could have immediate customer impacts.

Traditionally this has been achieved by setting fixed alarm limits around each incoming data point alerting operational staff if any pressure measurement is either too high or too low. However, given the variability of normal usage, setting these alarm setpoints has always proved problematic. Water companies are now turning to machine learning to identify patterns of usage to dynamically adjust the alarm profiles according to demographics, time of day, time of year, holiday periods, weather etc.

UK water companies are increasingly investing in more sophisticated methods of leak detection. One approach replicates the ability of humans to recognize the sound of a leak.

Companies are investing in large network of acoustic data loggers that take recordings from microphones attached to the drinking water network. This data is then made available to ML systems capable of interpreting the acoustic files.

#### 2.4.4. Internal sewer flooding

##### Classification: High data collection/high data utilization

Internal sewer flooding occurs in areas (frequently residential) where one, or more, properties are connected to a sewer pipe with a sub-optimal fall. Due to slow flow rates these pipes are prone to partial blockages that go unnoticed until it rains at which point contaminated water backs up flooding the properties.

Historically detecting these partial blockages to clear them ahead of bad weather has been notoriously difficult.

With recent advances in IIoT level sensors (devices that do not themselves encourage blocking and that are capable of transmitting data from a buried pipe), it is now possible to detect partial blockages. However, these devices are of little value without the associated machine learning required to differentiate genuine blockages from the noise generated by normal but intermittent use of the sewer.

#### 2.4.5. Combined sewer outlets

##### Classification: High data collection/low data utilization

It is recognized by the regulator (UK Environment Agency) that some sewer networks are expected to handle both effluent and rainwater (combined sewers) and it is unrealistic for treatment works to cope with the volumes of water associated with bad weather events. It is, therefore, necessary (under these conditions) to discharge a proportion of the untreated effluent into rivers. In practice this is managed by specially engineered chambers (Combined Sewer Outlets—CSOs) (Environment Agency, 2018).

The issue with CSOs is that a downstream blockage can cause them to discharge effluent outside of these permitted events, or when it is raining but at a level that the sewer should be able cope (these events are not sanctioned by the regulator). Therefore, CSO levels are constantly monitored so that teams can be dispatched to clear blockages.

To deliver this requires a large network of level sensors and data loggers, however the processing of this highly structured data into operational alerts is very simple.

#### 2.4.6. Condition-based monitoring

##### Classification: Low data collection/high data utilization

The supply of water and collection of waste is dependent on a large number of geographically dispersed pumping stations. To improve the reliability of these assets, while also reducing costs, water companies are increasingly investing in Condition Based Monitoring (CBM).

Unlike traditional approaches to maintenance (scheduled or fix on fail) CBM monitors the performance of the asset with maintenance only occurring when the asset falls outside a predefined performance envelope.

One of the most common parameters analyzed in pumping applications is vibration. However, for dispersed assets it is not practical to do this in real time—it being more common to acquire vibration data with a handheld device on a predefined rota (usually 4 weeks or longer). Although the data collected is highly structured it requires some sophisticated processing before it can be interpreted. Furthermore, machine learning is being increasingly deployed to augment the interpretation of the information (e.g., spectra and orbital plots) derived from the data.

The description of the data-driven water infrastructure systems highlights the inherent socio-technical nature of these cyber-physical systems, which is as such a complex system. Due to the narrow and distinct foci of IT and OT technical domains, in the following sections, we discuss how governing managerial frameworks struggle to convey normative (societal) challenges associated with data-driven water systems. We elaborate on the lack of focus on respecting the human right to drinking water, for current and future generations.

## 3. Conceptualizing water management with capabilities approach

After describing the complementary relationship of modern water management ecosystems with digital data-driven infrastructure, in the second part of this article, we analyze whether this relationship sufficiently tackles the challenges ahead of water management.

We identified earlier that the main future challenges of water management lie in the interrelated relationship between the (a) fulfillment of the human right to drinking water and hygiene; and (b) climate emergency that negatively influences the quality, availability, and access to drinking water. However, besides these points of departure, the overall purpose of water management goes beyond the narrow requirements of maintenance and protection. As all these aspects and the respective challenges are normative in nature, we seek to answer the following question: *to what extent does the datafication of water management systems sufficiently address the future challenges of water systems?* We utilize an adjusted version of Capabilities Approach (CA) for this normative analysis to answer the question.

We employ a theoretical approach to reflect on the normative underpinnings of data-driven water systems as critical infrastructure. The CA focuses on concrete and future capabilities, which—in this case, a data-driven critical infrastructure—should enable every human being to function. Through such a normative lens, we can better highlight the complex elements and prospects of a well-functioning data-driven water system as critical infrastructure for individual people now and in the future within a single analytical framework. For this, we will apply the CA developed by Nussbaum (2011) and Sen (1985, 1999, 2004, 2005) and further explicated by Robeyns (2017). CA aligns well with the human rights tradition (mentioned in Section 1) and the practical socio-technical challenges identified below.

CA builds upon a normative commitment to conceptualizing wellbeing in terms of capabilities and functionings (Robeyns and Byskov, 2020). Instead of being preoccupied with utilitarian measures (i.e., welfare) and available amounts of resources (i.e., a commodity-centered view), the CA shifts the focus onto capabilities—that facilitate a person's desired functionings (doings and beings) and contribute to her/his wellbeing. The strength of the CA lies in its ability to clearly conceptualize the capabilities preserved, maintained, or restored through the availability of water resources and water systems as CI. With the CA, the ultimate end for any interpersonal comparison lies in the realistically available options for choice, that is, capabilities (Kynch and Sen, 1983). Through this lens we will engage in an exploratory normative analysis of the current digitalized data-driven water management systems.

### 3.1. The challenges of water system management: Beyond current frameworks

The description of infrastructure by Bowker and Star (1999) as “visible upon break-down” (p. 35) refers not only to the phenomenon that we often take these systems for granted. In addition, the adjective *critical* infrastructure—defined as services, resources, people, and equipment (Aradau, 2010)—highlights the essential nature of these systems that need protection. While data on water resources and systems often serves conflict-resolving purposes (Muenger et al., 2020), just as water resources themselves (Schmeier et al., 2019), water data can also create conflict (Margesson, 1997). For instance, on the quality and interpretation of data, given that nations will likely differ in the way that they transform water data into information or how they judge the value of that data. On the path toward data-driven water systems and optimized water management, open questions around responsibility, fairness, and reliability of data-driven applications to water systems as critical infrastructure remain. In current affairs, attention is growing toward the protection of water systems as critical infrastructure, including subsystems of supply, sanitation, hygiene, and management. However, an important gap exists where not enough attention is being paid to the growing role and impact data has on water systems and water management that goes beyond the usual topics considered.

Michalec et al. (2021) highlight that, next to safety incidents and cybersecurity attacks, climate change-related events will continue to put additional pressure on the limited water management resources with far-reaching consequences. First, a change in regulatory incentives to increase cybersecurity, while capping the spending (and profit in the case of UK), can negatively affect available funds to tackle adverse events in water management, creating a disconnect with environmental governance (Michalec et al., 2021). Interviewed cybersecurity professionals did not consider issues of sustainability and environmental issues as critical at the time of the conduct of the study by Michalec et al. (2021). Similarly, the involvement of citizens in water planning and the broader cybersecurity was also not of concern (Michalec et al., 2021).

Second, another source of conflict emerges due to the different mindset between the water management industry stakeholders. Splitting the IT-OT perspectives creates tension between the importance of reliability, safety, and continuous availability (represented by OT experts); compared with practitioners focusing on privacy, security, accuracy, data recovery, etc. (represented by IT experts) (Michalec et al., 2021).

Third, the implementation of the EU Network and Information Systems (NIS) Directive (EC, 2016) by DCMS (2018) can potentially further exacerbate tensions beyond the water industry stakeholders to consumers. As a consumer-facing industry issues of business case, brand-protection, campaign for resources, asset management play out differently differently across the various IT-OT teams (Michalec et al., 2021). In particular, the connection of wide IIoT deployment to public interest, digital innovation, and regulation failed to ensure the benefits to citizens. Despite all the innovations in digital technologies, cybersecurity is still not widely considered an issue by the water consumers (Michalec et al., 2021).

Water systems have been studied from social (Makropoulos and Savić, 2019), technical (Giudicianni et al., 2020; Tuptuk et al., 2021), and socio-technical (De Haan et al., 2013; Kiparsky et al., 2013; Fuenfschilling and Truffer, 2016; Makropoulos and Savić, 2019) perspectives before. Whereas, technical perspectives focus on the physical infrastructure system, social perspectives take a closer look at the surrounding social system including elements such as organizations and institutions (Brown et al., 2009). Socio-technical systems require an approach that recognizes the interaction between people and technology (Fuenfschilling and Truffer, 2014; Pasmore et al., 2018). From the convergence of water management and digitalized data-driven infrastructure the increasing interconnectedness of the socio-technical domains is evident. The adjective data-driven implies that the collection, analysis, interpretation, and application of data inherently includes both technical and social aspects of water systems. Consequently, it is important to analyze the impact of data on human decisions, including the interactions and dependencies between social and technical systems (Ingildsen and Olsson, 2016), to design effective data-driven water systems. The study of Michalec et al. (2021) highlights that new regulatory frameworks do not sufficiently address the challenges associated with this socio-technical domain. In Table 1 we review three dominant management perspectives present in current discourse: Social Exchange Theory (Cropanzano and Mitchell, 2005; Cropanzano et al., 2017), Resource Based View (Newbert, 2006; Crook et al., 2008; Liang et al., 2010), and Transaction Cost Theory (Ghoshal and Moran, 1996; Geyskens et al., 2006; Schermann et al., 2016; Cuypers et al., 2021).

**Table 1:** Overview of the focus, impact, and challenges associated with three management theories applied to water management and infrastructure.

**Theories**	**Definition**	**Focus and impact**	**Challenges**
Social exchange theory	A sociological and psychological approach to understanding behavior in interactions between two parties who are implementing cost–benefit analyses to determine risks and benefits or the exchange of goods and thus are based on “voluntary actions of groups or individuals that are motivated by the returns they are expected to bring and typically in fact bring for others” (Blau, 2017, p. 91)	• Cost-benefit analysis • On risks and returns of exchange of goods • Trust and power • Social perspective	• Data quality and integrity • Design interactions with non-human unsupervised agents
Resource-based View	A managerial framework for understanding resources as valuable, rare, and relatively inimitable commodities that can be readily exploited by organizations to achieve sustainable competitive advantage	• Resources considered as rare, inimitable, exploitable • Achieving sustainable competitive advantage • Technical perspective	• Data security, easy exchange, and transmission • Efficiency and effectiveness of the whole system can be challenging • Closed systems, sustainability • Lack of clarity which features of data influence the functioning and outcomes of systems
Transaction-cost theory	Transaction cost theory argues that systems must decide to either make something themselves or buy it instead (Ouchi, 1980; Powell, 1990). The basic unit of analysis being the transaction, all transactions contain conflict, mutuality, and order between exchange actors and on the costs involved in those exchanges (Williamson, 1998, 2002). In exchanges where transaction-specific investments are high, the frequency of interaction between exchanging actors becomes limited, and the degree of behavioral uncertainty about the transaction increases, making exchanges within and between systems problematic. Transaction cost theory explains this phenomenon based on the opportunistic behavior of actors driven by self-interest and bounded rationality.	• Value creation or value-acquisition • Transactions, focusing on conflict, mutuality, order • Self-interest, bounded by rationality • Governance perspective	• High transaction costs stemming from quantity of formats, variety of licenses • Uncertainty of data implementation, use, analysis • Lack of innovations, and adequate governance models

Although management and organization sciences provide a plethora of theories and perspectives, we choose these three dominant management perspectives because: water systems in their development of becoming (more) data-driven; exchanges will increasingly become more artificial; data as a resource provides competitive advantage; and transactions in socio-technical systems demand governance forms and capabilities other than the traditional organization of mere human agents. We will now elaborate on three challenges water systems face in becoming data-driven.

Pre-digital water management systems were mostly reliant on social exchanges between human agents. Social exchanges are defined as “*voluntary actions of groups or individuals that are motivated by the returns they are expected to bring and typically in fact bring for others*” (Blau, 2017, p. 91). With the introduction of digitalized data-driven water management these interactions will increasingly become automated in workflows that partially or fully do not require human interaction. This will necessitate that we view interactions not only as social behaviors of exchange, but also as (in)voluntary processes based on the principles of reciprocity (Emerson, 1976) and FAIRness (Wilkinson et al., 2016; Collins et al., 2018; Stall et al., 2019); and overall, the social adaptations of system agents to technologies and data are required (Skjølsvold et al., 2015; Hoolohan et al., 2021).

**Figure 5:**
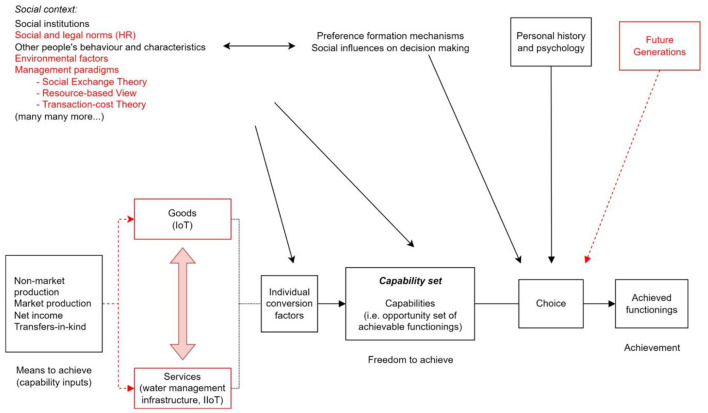
Capabilities Approach applied to data-driven water systems based on the graph by Robeyns (2005), p. 98. The highlighted sections in red are specifications and adjustments by the authors to the framework, applicable to data-driven water management systems policy. The figure also highlights the issue of the separation of goods and services, as a side-effect of data-driven water management, discussed below.

From the CA viewpoint (Figure 5) this changing environment alters the trust-relationship and social influences on decision-making and individual choice. While on the one hand big data may increase the insight of specific water management contexts, on the other hand the danger of non-validated, unsupervised, and untrustworthy data may break down parts of the water management system. We have seen that the slightly different objectives of IT-OT stakeholder teams may create tensions (Michalec et al., 2021) and with increased automation will further put the resilience of water management systems to test.

Another challenge relates to water and data as both a resource (i.e., something that needs protection) and a commodity (i.e., something that needs to be exchanged or traded) of water systems (Smith, 2009). We borrow from the resource-based view, a managerial framework for understanding resources as valuable, rare, and relatively inimitable commodities that organizations can readily exploit to achieve sustainable competitive advantage. Following the resource-based view, systems can be differentiated based on resource heterogeneity and resource immobility (Miller, 2019). As such, data from water systems can vary in their heterogeneity and immobility, which may carry implications for reproducing, combining, and transferring data. For example, suppose features of the data vary among various subsystems of the water system. In that case, such data heterogeneity can prevent data reproducibility, merger, or effective transfer, thus potentially diminishing its utility and even value as a resource. When data features significantly differ, systems must rely on different standards and formats, increasing their complexity.

The very nature of the easy exchange and transmission of digital data may hinder the proper implementation of strategies that aim at improving the efficiency and effectiveness of the whole system (Miller, 2019). This poses a security threat that can lead to the loss of a competitive advantage, which may result in the incentivization of stakeholders to protect their systems through obscurity (Scarfone et al., 2008). Consequently, water management regulatory frameworks need to address the right balance between overprotection and wasting resources, both in water, data, and human effort.

From the CA viewpoint, it can be argued that greater data transparency may address many technical issues in water management (e.g., leakage, environmental urgency), but the nature of this option needs to be explicitly established and validated (NWG, 2021; OfWat, 2021b; Yorkshire Water, 2021). Nevertheless, the dual-resource nature of data-driven water management systems will further exacerbate the issues noted about social exchanges and decision-making. This issue will apply to stakeholders within the water management ecosystem, as well as to consumers.

Finally, the transaction-cost theory highlights the evolving socio-technical challenge of data-driven water management. Transaction Cost Theory argues that systems must decide to either make something themselves or buy it instead (Ouchi, 1980; Powell, 1990). The basic unit of analysis being the transaction, all transactions contain conflict, mutuality, and order between exchange actors and on the costs involved in those exchanges (Williamson, 1998, 2002). In exchanges where transaction-specific investments are high, the frequency of interaction between exchanging actors becomes limited, and the degree of behavioral uncertainty about the transaction increases, making exchanges within and between systems problematic. Transaction cost theory explains this phenomenon based on the opportunistic behavior of actors driven by self-interest and bounded rationality. This applies not only to water- but also data exchange. For instance, the demonstrated quantity of formats and variety of licenses of (open) data in Figure 1 highlights the unnecessarily high transaction costs for actors in (open) data-driven water systems.

The fact of uncertainty regarding whether shared data can be implemented, used, or analyzed may—from the CA viewpoint—hinder further innovations in data-driven water systems and limit the capabilities of both water management systems as well as consumers (with spill-over effect to other industries, e.g., nuclear power Crellin, 2022).

## 4. The larger picture

Data-driven water infrastructure systems are complex socio-technical cyber-physical systems. As such, the societal and normative aspects of the current conceptualizations of data-driven water infrastructure systems require systematic reflection. The CA allows us to focus on these aspects in a deliberative process. This exploration will help determine the future of responsible and sustainable data-driven water infrastructure systems.

The CA lens provides a deeper insight into the overall normative challenges of digitalized data-driven water management systems. To highlight these, we briefly describe a scenario of the Climate Resilience Demonstrator (CReDo) film (O'Toole and Hayes, 2021), where critical national infrastructure (CNI) is controlled by data in the DT system:

During a stormy day, a grandfather and a son relax in the living room watching worrying news about an official flood warning in the UK due to torrential rain expected in the upcoming hours. The grandfather is breathing with the assistance of an oxygen respirator and is generally frail, with mobility difficulties. The grandson soon leaves his grandfather's house for a meeting. The weather worsens significantly, and after a couple of hours, the utilities stop working one after another because of the bad weather. The grandfather tries to turn on the electricity, on which his breathing machine, water pump, and other utilities depend. However, he is unsuccessful and soon loses consciousness. In the meantime, a DT engineering team operator asks a regional utilities provider to share their current data on the situation so that they can help ameliorate the emergency. After much persuasion, the utilities provider reluctantly shares their data. This enables the DT center operator to re-establish the proper functioning of electricity and other utilities in the region's households. In the meantime, the grandson returns to his grandfather, and is able to help him with the newly established utilities (Source: O'Toole and Hayes, 2021).

This scenario highlights the essential need for shared data for maintaining and protecting CNI across the digital infrastructure. These are not isolated networks. Instead, they are interconnected, intertwined, and interdependent. For instance, a sudden flood can disrupt telephone lines or electricity; a regional disruption in electricity can disrupt essential healthcare services, etc. The Hall (2022) report states that the negative effects of climate change on CNIs through interconnectedness and cross-dependencies can provide effective ways of protecting CNIs through better information management and data sharing. Based on the CA framework, with better insights into these complex systems, not only vulnerabilities of these systems can be addressed, but overall the quality of services should be increased, and waste of (not only financial) resources should be reduced. Through the normative lens, not only should the human right to drinking water be respected, but also the access to water and all dependent future functionings, which should also include future generations.

In the scenario above, the issue refers to missing multi-party data sharing agreements, which in Hall (2022) should lead to signing a data license (e.g., Data Exploration License) between asset owners, data hosting platform (e.g., Data and Analytics Facility for National Infrastructure), and other signatories. Such an agreement should facilitate smooth data exchange across CNI as service providers.

However, from Figure 5, one can argue that while the service providers struggle to collect relevant data across CNI, the large-scale introduction of consumer IoT devices into homes may provide additional data for the maintenance and protection of CNI from the IT and OT perspectives. While the slow pace of digitalization of CNI services struggles with sufficient data collection, the wide availability and deployment of consumer IoT devices create a gap between services and goods. This might harm overall service availability, hindering the human rights to drinking water and sewage in the long term. Consequently, CNI's resilience to the effects of climate change may be compromised, contributing to the lack of functionings and capabilities of citizens.

Currently, no governance or regulatory frameworks exist that allow citizens to provide their data for the pro bono protection and maintenance of CNI. Some federated efforts exist in cybersecurity domains [e.g., honeypot-as-a-service (HaaS)^4^, CrowdSec^5^]. However, it would be desirable to extend these to the OT domain too. For example, a better insight into a potential water leakage at a consumer level may prevent the development of larger issues later, contributing to agile problem management, increased robustness and resilience, and decreased waste in water systems. Similarly, a report of a cybersecurity incident may prevent large-scale vulnerabilities of the whole CNI system.

## 5. Conclusion

Water is essential for life, and access to it is a widely accepted, basic, and universal human right. Water systems are, therefore, critical infrastructure with Information and Operational Technologies involved. The digital transformation of our societies includes the datafication of water resources and the digitalization of water systems, which introduces many new complexities into these evolving socio-technical systems. Viewing these systems through the lenses of the social exchange theory, resource-based view, and transaction cost theory has revealed their limitations. Viewing these complexities through the lens of CA highlighted that the focus on present-future capabilities can incorporate broader normative claims (e.g., human rights; climate emergency) and settle decisions for future courses of action. The multidisciplinary exploration of several key challenges encountered with data-driven water systems as critical infrastructure has determined potential paths to address these challenges, and improve and optimize data-driven water infrastructures. These include putting in place governance that implements the standardization of data features (e.g., FAIRness); the development of clear regulation, codes of conduct, and best practices within industries; and the facilitation of data sharing that more widely engages citizens as well as researchers and which fosters innovation and the healthy growth of economies and communities, in addition to helping realize actual freedoms and capabilities for individual persons. These are just some of the possible routes to optimizing data-driven water systems as critical infrastructure to responsibly and justly use and share water and data on water and water systems to ensure the wellbeing of all persons, other species, and the natural world on our blue planet, now and in the future.

## Data availability statement

Publicly available datasets were analyzed in this study. This data can be found here: https://data.europa.eu/en.

## Author contributions

All authors listed have made a substantial, direct, and intellectual contribution to the work and approved it for publication.

## Footnotes

^1^https://data.europa.eu. Further information on the EU Water landscape can be found in EurEau (2021).

^2^Despite the phasing out of PSTN (Ofcom, 2019) it is included here as it is a convenient measure of the lower bound of sophistication.

^3^DaaS is closely aligned with IIoT where large datasets are procured *via* a third party, even if that third party attaches their monitoring equipment to the company's own assets to acquire the data.

^4^ https://haas.nic.cz/

^5^ https://www.crowdsec.net/
